# Small Colon Faecalith with Large Colon Displacement in Ten Cases (2015–2023): A Detailed Case Description and Literature Review

**DOI:** 10.3390/ani14020262

**Published:** 2024-01-15

**Authors:** Nicola Scilimati, Anna Cerullo, Sara Nannarone, Rodolfo Gialletti, Gessica Giusto, Alice Bertoletti

**Affiliations:** 1 Department of Veterinary Medicine, University of Perugia, 06126 Perugia, Italy; nicola.scilimati@yahoo.it (N.S.); sara.nannarone@unipg.it (S.N.); alicebertoletti1@gmail.com (A.B.); 2 Department of Veterinary Sciences, University of Turin, 10095 Grugliasco, Italy; anna.cerullo@unito.it (A.C.); gessica.giusto@unito.it (G.G.)

**Keywords:** colic, faecalith, large colon volvulus, right dorsal displacement, small colon focal impaction

## Abstract

**Simple Summary:**

Impactions are the most common issue affecting the small colon in horses, particularly in miniature horses and ponies. Risk factors for small colon faecalith include poor dentition, reduced water consumption, parasite damage, or poor-quality hay. Abdominal pain can range from mild to severe due to intraluminal gas distention orally to the impaction site. Previously, large colon tympany has been identified as a predisposing factor for large colon displacement or volvulus. The aim of this study was to describe the history, clinical and surgical features, and outcome in 10 cases which underwent exploratory laparotomy, where a diagnosis of small colon faecalith concomitant with large colon volvulus or right dorsal displacement was reached. In all clinical cases, the small colon faecalith was determined to be the primary lesion during celiotomy, underscoring the importance of a thorough examination of the small colon tract when diagnosing large colon displacement or volvulus during exploratory laparotomy.

**Abstract:**

Small colon impaction can result in accumulation of ingesta, gas, and fluid orally to the impaction site in horses. Large colon tympany, which is caused by ingesta fermentation, can be a predisposing factor for intestinal displacement. The aim of this study is to report the history, clinical, and surgical findings of horses and ponies referred for abdominal pain not responsive to drugs where a right dorsal displacement (RDD) or large colon volvulus (LCV), together with a small colon faecalith (SCF), were diagnosed during surgery. This study included a total of five horses and five ponies. Based on clinical features, ultrasonographic examination, and rectal palpation, an initial suspected diagnosis of RDD, LCV or severe large colon and caecum distension was made in all clinical cases. Due to the lack of response to medical treatment or worsening of colic symptoms, surgery was performed in all horses: diagnosis of RDD or LCV was made and a SCF was incidentally detected in all cases. While exploratory laparotomy was carried out in all the patients for the presence of a large colon issue, it was probably subsequent to an obstruction of the small colon caused by the presence of an SCF, which is generally difficult to diagnose. This study reported the presence of SCF as a possible cause of secondary RDD or LCV in horses and ponies that, to the authors’ knowledge, has never been reported.

## 1. Introduction

Large colon displacement (LCD) is one of the leading causes of colic in horses, with surgical treatment rates ranging from 19% to 36% [1,2,3,4,5]. Several risk factors have been identified in the development of LCD, including changes in diet, exercise, management, and quidding behaviour [6]. In a prior study by Lopes and colleagues in 2004, it was suggested that alterations in colonic contents resulting from a high-grain diet might lead to right dorsal displacement (RDD) or large colon volvulus (LCV) [7]. In the case of RDD, the large colon moves around the caecal base and part of it is positioned between the caecum and the right abdominal wall [6]. The LCV is the rotation of the colon across or around its mesentery and could be in a clockwise or counterclockwise direction [6]. During rectal palpation (RP), suspicion of RDD or volvulus of the large colon may arise. While RP alone may not definitively diagnose these conditions, it can indicate signs such as tympany, impaction, or abnormal positioning of the large colon [8]. In cases of RDD the teniae and the large colon may be palpable above the pelvic rim, extending horizontally from the right cranial abdomen to the left caudal abdomen [8]. Tympany, induced by fermentation of residual static ingesta due to motility alterations or impaction, appears to be a predisposing factor for intestinal displacement [7,9,10]. As such, any condition leading to gas accumulation may pose a risk factor for the development of LCD or LCV. While LCD or LCV might be the primary reason for equine referral, these are likely to be secondary conditions stemming from an underlying problem, such as small colon impaction, which may require a more investigative diagnosis [11]. Small colon impaction is typically linked to mild colic pain, which can exacerbate due to oral distension of the intestine caused by the obstruction [12,13]. For this reason, initial signs of abdominal pain may go unnoticed. Abdominal distension may be severe in horses with complete obstruction of the small colon lumen, leading to an increase in heart rate and abdominal pain in those horses requiring surgery [13]. Faecal impaction is the prevalent condition affecting the small colon in horses (34% of small colon disease) [11,13]. However, this intestinal tract can be occasionally focally obstructed by a faecalith, enterolith, phytobezoar, and trichobezoar [14,15]. Faecaliths are made up of concretions of dry and inspissated faecal material, characterised by a more distinct shape than general feed impaction [15]. Small colon faecaliths (SCF) represent one of the most common obstructions among focal impactions [11,14,15,16]. According to Dart and colleagues (1992), American Miniature Horses, Arabians, ponies, and horses aged over 15 years are predisposed to this condition [17], although other studies have not identified breed or age as predisposing factors [18,19,20,21,22,23]. Several risk factors for an SCF, including the ingestion of poor-quality hay, inadequate dentition, insufficient mastication, intestinal motility disorders, parasite damage, and reduced water intake, have been reported [12,22]. Initial clinical signs of SCF are often nonspecific and vague, with symptoms like mild abdominal pain, lethargy, decreased faecal passage, or diarrhoea [11,23,24]. Additionally, transabdominal ultrasonography (US) and RP findings are typically not helpful in diagnosing a specific site of small colon obstruction such as SCF, attributable to the lack of specificity [11]. Due to the complexity of making a definitive diagnosis of SCF, it may sometimes be unappreciated, resulting in intestinal distension and ultimately in the exacerbation of colic symptoms. The lack of the progression of material within the intestinal lumen leads to an increase in intraluminal gas and fluid [11,12]. Several studies have reported that large colon tympany is a predisposing factor for LCD or LCV [7,8,9,25]. Strangulating LCV can significantly impact the postoperative course of a patient, increasing the risk of postoperative complications and leading to an unfavourable prognosis respect to horses diagnosed with LCD or tympany [26]. Therefore, prompt surgical treatment may be crucial, especially when there is suspicion that LCD is associated with a concomitant disease like SCF. Although the previous literature has indicated that small colon impaction can predispose a horse to LCD [12,20,24], to the best of authors’ knowledge, cases of SCF associated with RDD or LCV have not been previously documented. The aim of this study is to describe the history, clinical findings, and surgical findings of horses with concurrent SCF and RDD or LCV.

## 2. Materials and Methods

### Animals

Medical records of all weanling foals, adult horses, and ponies that underwent exploratory laparotomy at the veterinary teaching hospitals (VTHs) of Perugia and Turin (Italy) from January 2015 to April 2023 were reviewed. Cases were included if LCD or LCV together with SCF were identified at exploratory laparotomy. The recorded data included age, breed, body weight, and sex (gelding, female, or male). Details of the surgical findings were reported for each case. Postoperative complications were defined as any clinical abnormalities detected during hospitalisation. Short-term follow-up was determined as survival until discharge from the VTHs. Long-term follow-up was defined as the period extending from discharge to June 2023, which was the date when a telephone interview with the owners was carried out.

## 3. Results

A total of 640 horses underwent exploratory laparotomy between January 2015 and April 2023. A LCV without concomitant lesions was present in 108/640 cases, a LCD (RDD or nephrosplenic entrapment) without concomitant lesions was detected in 158/640 horses. Furthermore, an SCF without concomitant lesions was found only in 22/640 cases.

Five horses and five ponies out of total 640 cases met the inclusion criteria for this study. Their ages ranged from 20 days to 29 years and they had a mean weight of 321.5 ± 204.4 kg. There were four geldings, four females, and two males. The breeds consisted of Quarter Horse (*n* = 2), Friesian (*n* = 1), Murgese (*n* = 1), Shetland (*n* = 1), Shire (*n* = 1), and unknown breed ponies (*n* = 4).

The history, clinical findings, and surgical results of five horses and five ponies referred to the VTHs due to colic pain unresponsive to medical treatment were reported. Horses initially presented with mild to moderate colic pain, and after clinical examination and rectal palpation in the field, referring veterinarians suspected a large colon issue; therefore, medical treatment was instituted. Due to the worsening of the clinical conditions despite analgesics, horses were referred to the VTHs. At arrival, all horses underwent a thorough clinical examination; all the patients were confirmed to be unresponsive to analgesics, and surgical treatment was then recommended for each case. Due to their small size, ponies and foals did not undergo RP. Signalment and the clinical status upon arrival of each horse are documented in Table 1.

### 3.1. Case Descriptions

Upon arrival, Case 1 presented with severe abdominal pain that was unresponsive to analgesics. A diagnosis of LCD was suspected at RP. Due to the severity of abdominal pain, celiotomy was recommended. During surgery, a diagnosis of RRD was made; the large colon was exteriorised, and a pelvic flexure enterotomy was performed after displacement resolution, allowing for the evacuation of ingesta. While systematically exploring the abdominal cavity, an SCF was also noticed. The mass felt firm and non-reducible to palpation, necessitating an enterotomy of the small colon to allow the SCF to be cleared. The enterotomy was performed at the antimesenteric band of the small colon and it was closed with a simple continuous pattern followed by a continuous Cushing pattern (2-0 USP Polydioxanone). Abdominal lavage with sterile saline was followed at the end of the surgery by standard abdominal closure in a double layer simple continuous pattern at the level of the muscle fascia (linea alba) and at the level of the skin. After surgery, the horse developed laminitis and hyperlipemia, both of which were successfully treated and resolved before discharge on the 10th day after the surgery.

Case 2 was referred for colic pain of 1 day in duration that was managed in the field with analgesics. After no response to medical treatment, the horse was referred to the hospital. Gas distension of the large colon was detected at RP together with a transverse band, painful on traction. Abdominal US revealed a distended large colon with an increased thickness of the large colonic wall on the right side. Due to suspected RDD, and the worsening of clinical conditions, celiotomy was recommended, and the diagnosis was confirmed during surgery. The displacement was successfully resolved following exteriorisation of the large colon, and a pelvic flexure enterotomy was performed. Additionally, during the evaluation of the small colon, a focal impaction caused by a faecalith was incidentally discovered. A small colon enterotomy was performed similarly to the previous case. Standard abdominal wall closure was performed, and the horse recovered uneventfully from anaesthesia. No complications were reported during hospitalisation and the horse was discharged after 10 days.

Case 3 was referred for colic pain of more than 24 h in duration. Upon arrival, a transverse band in the abdomen with moderate gas distension of the large colon was identified during RP, leading to a provisional diagnosis of RDD. Abdominal US was not conducted. After initial medical treatment, there was a slight increase in abdominal pain, and surgical treatment was recommended as for previous cases. A ventral midline laparotomy was performed, confirming RDD, which was then corrected, and a pelvic flexure enterotomy was carried out. Intraoperative palpation of the small colon revealed a focal impaction due to a faecalith. The SCF appeared to be reducible, and the obstruction was resolved through extraluminal manipulation of the intestinal tract until rectal expulsion was achieved. Abdominal closure was performed as for previous cases. During the hospitalisation, the horse developed laminitis and hyperlipemia, both of which were effectively resolved before the horse was discharged 10 days post-surgery.

Case 4 was referred for colic pain of 3 days in duration managed with analgesics and intravenous fluid therapy. Passage of a nasogastric tube resulted in 9 litres of net reflux. RP was not performed due to the small size of the animal. Abdominal US revealed a mild increase in anechoic peritoneal fluid, gas distention of the large colon, and the presence of some distended small intestinal loops. Based on clinical findings, surgical treatment was advised. The pony was intraoperatively euthanised due to the presence of a rupture involving the right dorsal colon which was displaced.

**Figure 1:**
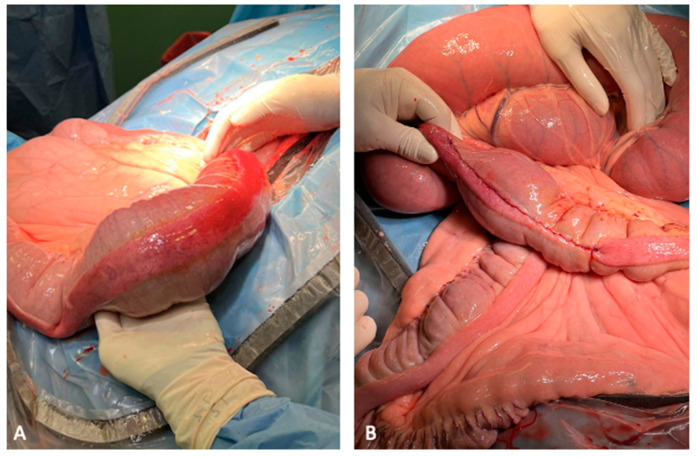
Faecalith, small colon, 17-year-old Murgese gelding. (**A**) Faecalith detected during exteriorisation of the small colon from the abdomen; note the moderate hyperaemia of the small colon serosa at the level of the faecalith. (**B**) The enterotomy at the antimesenteric band of the small colon was closed with a two-layer closure using a full-thickness simple-continuous pattern followed by a seromuscular inverting pattern (Cushing) with 2-0 USP polydioxanone.

Case 5 was referred for colic pain of approximately 36 h in duration. The clinical progression of symptoms was similar to previous cases with the presence of mild gastric and large colon distension. During US examination, ectasic vessels in the right middle hemiabdomen were identified, raising suspicion of RDD or LCV. As abdominal pain slightly increased, surgical treatment was recommended. A ventral midline laparotomy was performed, confirming the diagnosis of RDD. The large colon was relocated and exteriorised allowing enterotomy at the pelvic flexure. During the exteriorisation of the small colon, an SCF was also observed (Figure 1A). A small colon enterotomy was performed (Figure 1B). The horse was discharged without complications after 15 days.

**Figure 2:**
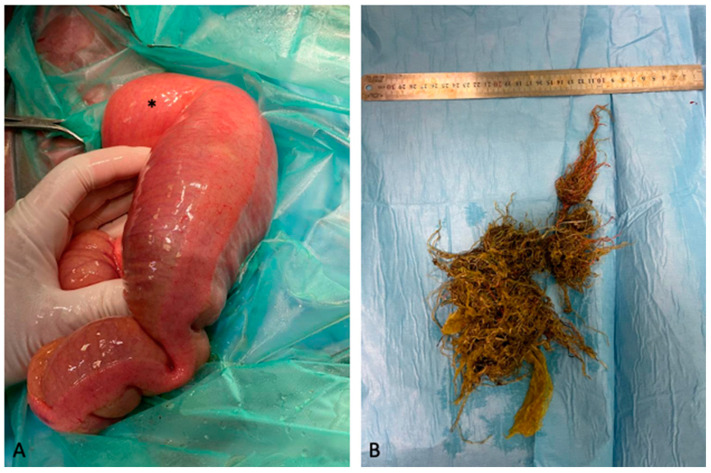
Faecalith, small colon, 8-month-old weanling Quarter Horse. (**A**) Small colon faecalith found incidentally during laparotomy after correction of a right dorsal displacement of the large colon in this colt; note the gas distention of the small colon proximally to the obstruction site (asterisk). (**B**) Hay net found inside the faecalith after its crumbling.

Case 6 was a Quarter Horse weanling colt referred for colic pain which had been ongoing for 24 h and presenting with marked dullness and severe abdominal distension. Due to its small size, RP was not performed. Abdominal US revealed the presence of gas in the large colon and reduced evidence of ventral large colon sacculations. Furthermore, a moderate increase in the amount of anechoic peritoneal fluid was detected in the right hemiabdomen. Even if no ectasic vessels were detected in the right hemiabdomen, a RDD was suspected. Based on clinical presentation and the duration of colic symptoms, a celiotomy was immediately recommended. Subsequently, a ventral midline laparotomy was performed, RDD was diagnosed and corrected, followed by pelvic flexure enterotomy. Also, in this case, after the exteriorisation of the small colon, an SCF was identified (Figure 2A). An antimesenteric enterotomy was performed as for previous cases. After removal, the SCF was crumbled and a hay net inside the faecal mass was identified (Figure 2B). After surgery, the horse showed pyrexia and developed right jugular vein thrombophlebitis. Both conditions were treated and resolved before the horse was discharged 10 days after surgery.

Case 7 was referred for severe colic pain lasting for 5 h. During RP, an impacted and axially displaced pelvic flexure was identified. The abdominal US showed mildly ectasic vessels in the right hemiabdomen. The pony was initially treated medically since it he responded to sedatives and scopolamine, but it underwent surgery the day after admission due to significantly increased abdominal pain. At ventral midline laparotomy, a 180° counterclockwise LCV was detected and corrected through reverse rotation. Additionally, a large colon impaction was discovered, necessitating a pelvic flexure enterotomy. During the evaluation and exteriorisation of the small colon, an SCF was noted, which was resolved through a small colon enterotomy. No complications developed during hospitalisation, and the horse was discharged after 13 days.

Case 8 was the same horse as Case 7 that was referred again for colic syndrome three years later. Upon arrival, it exhibited moderate abdominal pain. RP revealed severe distension of the large colon on the right side of the abdomen. The abdominal US showed gas distention in the large colon and caecum. Based on these clinical and US findings, a diagnosis of recurrent SCF associated with large colon tympany or RDD was suspected, leading to the recommendation of laparotomy. During the procedure, RDD was identified and corrected, and a pelvic flexure enterotomy was performed. Following the exteriorisation of the small colon, a recurrence of an SCF was discovered. The faecalith appeared reducible, and obstruction was resolved through extraluminal massage, similarly to the approach used in Case 3. The pony was discharged after 10 days without complications. Approximately two months later, the pony was found dead in the field; no necropsy was performed.

Case 9 was referred for colic pain persisting for more than 30 h that was unresponsive to analgesics. Upon arrival, the pony was still partially sedated and consequently showing only mild colic pain. RP was not performed at arrival due to the small size of the animal. Upon US examination, gas was observed in the large colon, and there was reduced evidence of ventral large colon sacculations on both left and right sides of the abdomen. Due to the presence of marked dullness and increasing colic pain a few hours later, the pony underwent a ventral midline laparotomy, during which RDD was identified, and a pelvic flexure enterotomy was performed. While evaluating and exteriorising the small colon, an SCF involving its proximal portion was noted and resolved through extraluminal massage, since it was reducible. During the hospitalisation, the pony presented hyperlipemia, which was successfully treated before discharge 7 days after surgery.

The last case (Case 10) was a 20-day-old foal admitted to the VTH for mild colic pain lasting for 12 h, which had been weaned a few days before referral. RP was not performed due to the small size of the foal. Abdominal pain moderately increased a few hours after admission, and surgical treatment was recommended. Following abdominal US examination, gas in the large colon and reduced evidence of ventral large colon sacculations were detected. A ventral midline laparotomy was performed and a 180° counterclockwise LCV was found and corrected by reverse rotation. After the exteriorisation of the small colon, an SCF was also detected and an enterotomy was made as previously described. During hospitalisation, surgical site infection occurred and multiple episodes of pyrexia were recorded. The foal was discharged after 11 days.

### 3.2. Outcome

All the horses and ponies that recovered from anaesthesia were discharged from the VTHs after a mean of 11 days. Updates on the clinical status of the patients after discharge were collected in June 2023. Details on postoperative complications and short- and long-term follow-up are summarised in Table 2.

## 4. Discussion

The current literature has widely described small colon impaction in the horse, due to the presence of enterolith, faecalith, or extensive faecal impaction [16,19,27], but faecal obstruction is among the most common causes of this disease [13,17,20,21]. Several studies hypothesised the possibility that an SCF could cause large colon tympany and LCD [11,12], but an association between this condition and LCD or LCV has been rarely reported [16] and there are currently no studies that document the presence of small colon focal impaction caused by faecalith in association with RDD or LCV. According to the literature, an SCF has usually been found in horses younger than 1 year and older than 15 years and in ponies [10,11,13], except for one recorded case in an 8-year-old Friesian horse. Other risk factors have been reported for this disease, such as poor-quality diet or poor dentition [13,20]. Presence of foreign material in an SCF was detected only in a weanling colt, and this can be explained by the less discriminating eating habits of young horses [11].

The diagnosis of focal impaction of the small colon can be difficult for several reasons. In the early stages of the disease, mild to moderate or intermittent pain is described [11,12,28] and impaction of the small colon may be associated with non-specific clinical signs [23,24]. Small colon impaction may be preceded by frequent minor colic episodes caused by intermittent large colon or transverse colon impaction, which often resolve with medical treatment [11]. The progression of clinical signs is slow, and the average time between the onset of severe colic pain and admission to a surgical facility usually exceeds 24 h [29]. This is in line with the current results, since, in most horses, clinical signs of colic began more than 24 h before surgery.

The presence of diarrhoea is another sign that can hinder a small colon impaction diagnosis [23,24]. It can be secondary to gastrointestinal stasis and inflammation related to the small colon impaction [21] or because the impaction only allows the passage of liquid material but not of the solid component [11,23,24]. However, it was not observed in any horses included in the study.

Diffuse impaction of the small colon is easily diagnosed upon RP, while the SCF may not be palpated if it affects the oral tract of the descending (small colon) or the transverse colon, or where there is concomitant gas distension of small colon, large colon, and caecum [11,12]. In addition, transabdominal US is not associated with any specific findings, unlike other diseases [12]. All these factors can lead to the underestimation of the condition and failure to institute the correct treatment: if not surgically addressed, small colon focal impaction due to a faecalith can cause progressive compression of the intestinal wall, with its subsequent necrosis and resultant intestinal rupture with a poor prognosis [12].

In all horses included in this report, an initial diagnosis of LCD was made, based on clinical, US, and RP examination findings. Medical treatment for LCD had been initiated but surgery was performed due to the worsening of colic symptoms. During surgery, diagnosis of RDD or LCV was confirmed and an SCF was found on examination of the small colon. Although surgical treatment was advised and performed in all cases for the presence of RDD or LCV, it is likely that these diseases were secondary to a small colon obstruction caused by the presence of an SCF.

In this study, all animals underwent enterotomy of the pelvic flexure before diagnosing an SCF, due to the significant amount of ingesta detected in the large colon. Evacuation of faecal material through large colon enterotomy is strongly recommended in horses with a small colon impaction to reduce its recurrence in the early postoperative period due to inflamed and oedematous small colon wall [11]. Treatment of impaction commonly includes aggressive enteral and parenteral fluid administration [12,29,30], but it was reported that aggressive enteral fluid administration to treat colon impaction could predispose a horse to LCV, resulting in a worsening of colic pain [31]. Furthermore, impaction is sometimes difficult to hydrate, and surgical intervention is necessary to prevent necrosis of the intestinal wall [11].

Several studies reported that the presence of small colon obstruction may predispose a horse to gas, liquid, and ingesta accumulation in the large colon [11,12,19,20]. In cases of intermittent obstructions, there may be an alteration of the normal intestinal physiology and microbiota [21,32,33]. All these conditions have been recognised as risk factors for the development of LCD [10,33,34]. However, although some studies hypothesised an association between small colon obstruction and a secondary LCD [11,20], to the best of the authors’ knowledge, there are no studies reporting an association between the SCF and RDD or LCV and, based on clinical findings, a diagnosis of RDD or LCV was suspected in all cases. Hanson and Schumacher (2021) reported that the correct position of the large colon should be always evaluated when a celiotomy for small colon impaction is performed, because LCD or LCV may happen secondary to gas distension orally to the impaction [11,12]. In this study, the SCF was an associated finding found at exploratory laparotomy after correction of the RDD or LCV in all horses.

The small number of cases represent the main limitations of this study. Furthermore, the authors could not define the chronology of the occurrence of the concomitant pathologies detected at laparotomy.

## 5. Conclusions

In conclusion, to the authors’ knowledge, a study showing an SCF associated with secondary RDD or LCV has never been reported. Based on the results of this study, the authors encourage surgeons to consider the possibility of SCF when LCD or LCV is diagnosed at surgery and that a systematic, complete abdominal examination at laparotomy is always warranted.

## Figures and Tables

**Table 1:** Data on signalment and clinical status at arrival of horses and ponies referred for suspected large colon displacement to the veterinary teaching hospitals of Perugia and Turin (Italy) between January 2015 and April 2023. y = years, m = months, d = days. * Case 8 is the same as Case 7.

Patient ID	Age	Breed	Sex	Weight (Kg)	HR (bpm)	RR (bpm)	CRT (sec)	PCV (%)	TP (g/dL)	Blood Lactate (mmol/L)	Intestinal Motility	Net Reflux (L)
1	18 y	Quarter Horse	Female	490	60	30	3	38	7.2	-	Absent	5
2	8 y	Friesian	Female	630	56	20	2	39	5.2	-	Absent	0
3	21 y	Unknown (pony)	Gelding	260	40	20	2	27	6.0	3.3	Absent	0
4	7 y	Unknown (pony)	Female	120	60	24	2	30	6.0	3.3	Absent	9
5	17 y	Murgese	Gelding	630	60	24	2	35	7.5	3.3	Decreased	-
6	8 m	Quarter Horse	Male	205	84	24	2	34	6.4	3.8	Unremarkable	0
7	26 y	Unknown (pony)	Gelding	340	60	28	<2	30	6.8	0.9	Decreased	-
8 *	29 y	Unknown (pony)	Gelding	340	52	24	2.5	31	6.3	2.3	Decreased	0
9	16 y	Shetland	Female	80.5	36	20	2	24	6.7	3.1	Decreased	-
10	20 d	Shire	Male	180	28	40	2	30	6.8	1	Decreased	-

**Table 2:** Details on postoperative complications and short- and long-term follow-up of the cases referred at the veterinary teaching hospitals of Perugia and Turin (Italy) from January 2015 to May 2023, which underwent ventral laparotomy for large colon displacement or large colon volvulus and small colon faecalith. * Case 8 is the same as Case 7.

Patient ID	Postoperative Complications	Short-Term Outcome	Long-Term Outcome; Duration
1	Laminitis–Hyperlipemia	Discharged	Lost at follow-up
2	Diarrhoea–Pyrexia	Discharged	No complications; 12 months
3	Laminitis–Hyperlipemia	Discharged	No complications; 12 months
5	None	Discharged	Incisional infection; 6 months
6	Thrombophlebitis–Pyrexia	Discharged	Incisional infection; 8 months
7	None	Discharged	Recurrence of problem; 13 months
8 *	None	Discharged	Died 2 months after discharge
9	Hyperlipemia	Discharged	No complications; 40 days
10	Surgical site infection–Pyrexia	Discharged	Lost at follow-up

## Data Availability

All of the data generated or analyzed during this study are included in this published article.
